# Biodegradation potential of cyano-based ionic liquid anions in a culture of *Cupriavidus* spp. and their in vitro enzymatic hydrolysis by nitrile hydratase

**DOI:** 10.1007/s11356-013-2341-2

**Published:** 2013-12-13

**Authors:** Jennifer Neumann, Magdalena Pawlik, Dieter Bryniok, Jorg Thöming, Stefan Stolte

**Affiliations:** 1 Department 3 Sustainable Chemistry, UFT - Centre for Environmental Research and Sustainable Technology, University of Bremen, Leobener Straße, 28359 Bremen, Germany; 2 Polish AGH University of Science and Technology, A. Mickiewicza 30 Ave. 30-059, Kraków, Poland; 3 Department of Environmental Biotechnology and Bioprocess Engineering, Fraunhofer Institute for Interfacial Engineering and Biotechnology IGB, Nobelstraße 12, 70569 Stuttgart, Germany; 4 Department 4 Chemical Engineering - Recovery and Recycling, UFT - Centre for Environmental Research and Sustainable Technology, University of Bremen, Leobener Straße, 28359 Bremen, Germany; 5 Department of Environmental Analysis, University of Gdańsk, ul. Sobieskiego 18/19, 80-952 Gdańsk, Poland

**Keywords:** Biodegradation, Ionic liquids, Cyano groups, Axenic culture, Biological treatment, Hazard assessment, *Cupriavidus* spp., Nitrile hydratase, Nitrilase

## Abstract

Biodegradation tests with bacteria from activated sludge revealed the probable persistence of cyano-based ionic liquid anions when these leave waste water treatment plants. A possible biological treatment using bacteria capable of biodegrading similar compounds, namely cyanide and cyano-complexes, was therefore examined. With these bacteria from the genera *Cupriavidus*, the ionic liquid anions B(CN)_4_^−^, C(CN)_3_^−^, N(CN)_2_^−^ combined with alkaline cations were tested in different growth media using ion chromatography for the examination of their primary biodegradability. However, no enhanced biodegradability of the tested cyano-based ionic liquids was observed. Therefore, an in vitro enzymatic hydrolysis test was additionally run showing that all tested ionic liquid (IL) anions can be hydrolysed to their corresponding amides by nitrile hydratase, but not by nitrilase under the experimental conditions. The biological stability of the cyano-based anions is an advantage in technological application, but the occurrence of enzymes that are able to hydrolyse the parent compound gives a new perspective on future cyano-based IL anion treatment.

## Introduction

Ionic liquids (ILs) have become an innovative substance group for industry and research purposes. The generic property of being ionic and liquid below a temperature of 100 °C stems mainly from the combination of asymmetric organic cations and anions. The combinability of their different components has led to a vast number of chemicals with different physico-chemical properties. ILs are most commonly used because of their very good solvent and catalytic properties (Welton 1999; Yue et al. 2011). Low vapour pressure and non-flammability are additional common key properties that make for improved operational safety in comparison to conventional solvents. This combination of IL properties has advantages in the fields of chemical synthesis and catalysis (Olivier-Bourbigou et al. 2010; Sheldon 2005), liquid–liquid extraction and enzyme stabilisation (Dreyer and Kragl 2008), and electrochemical (Liu and Pan 2011) and analytical applications (Berthod et al. 2008) in that the relevant processes are more effective and safer (Kokorin 2011). However, the environmental risks of IL disposal have not yet been fully examined. Looking at the main source for ILs into the environment, it is probable that ILs enter the environment via waste water treatment plants when physico-chemical properties and applications of ILs are taken into account (Siedlecka et al. 2010). Adsorption on solid surfaces is also possible as a second source, especially for lipophilic ILs. As non-volatile substances a direct contamination of air is unlikely. Therefore, biodegradability tests using activated sludge from waste water treatment plants have been conducted for a range of ILs with varying results from being low to readily biodegradable, depending on the structural composition of the IL (Stolte et al. 2011). These tests have shown IL anions containing cyano groups to be non-biodegradable (Neumann et al. 2012) and hydrolytically stable under environmental relevant conditions (Steudte et al. 2012). Only N(CN)_2_^−^ and C(CN)_3_^−^ could be hydrolysed at very strong acidic (pH 1) and basic conditions (pH 13). The technological relevance of the tested IL anions comes from their potential application as alternatives for the commonly used fluorinated anions bis(trifluoromethylsulphonyl)amide (CF_3_SO_2_)_2_ N^−^ (BTA) and trifluorotris(pentafluoroethyl)phosphate (C_2_F_5_)_3_PF_3_^−^ (FAP). The fluorinated anions lower the melting point and the viscosity of the IL, increase its hydrophobicity, and widen the electrochemical window for a better technological applicability (Xue et al. 2006). However, these fluorinated ILs are also highly refractive towards abiotic and biotic degradation processes (Ignat'ev et al. 2005; Neumann et al. 2012; Steudte et al. 2012). The cyano-based IL anions have a similar effect to the physico-chemical properties of the IL for technological application but were shown to be even more effective than the standard fluorinated BTA containing IL in CO_2_/N_2_ separation by supported IL membranes (SILMs) (Mahurin et al. 2010). N(CN)_2_^−^ was also just recently been studied for a usage in lithium batteries (Yoon et al. 2013). Furthermore, this anion was reported to be a good counterion of IL solvents and catalysts for the processing of alcohols and sugars (Forsyth et al. 2002). Cyano-based ILs have further been reviewed as solvents for liquid–liquid extraction of aromatic hydrocarbons in place of the conventionally used sulfolanes (Meindersma and Haan 2012). Additionally, C(CN)_3_^−^ and B(CN)_4_^−^ are of high interest in renewable energy production as electrolyte additives in dye-sensitised solar cells (Kuang et al. 2006; Marszalek et al. 2011).

**Fig. 1:**
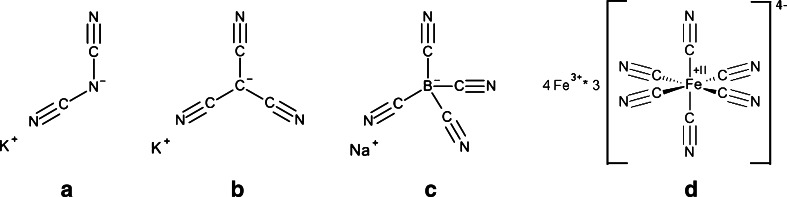
Molecular structures of the ionic liquids used in this study: **a** sodium dicyanoamide Na N(CN)_2_, **b** potassium tricyanomethanide K C(CN)_3_, and **c** potassium tetracyanoborate K B(CN)_4_ and the reference substance Prussian blue (PB, Fe(III)_4_[Fe(II)(CN)_6_]_3_)

Since the cyano-based anions, K B(CN)_4_, K C(CN)_3_ and Na N(CN)_2_ have shown not to be biodegradable under aerobic and denitrifying conditions (Neumann et al. 2012); a mix of two bacteria strains that are capable to biodegrade one of the most stable cyano complexes, Prussian blue (PB) Fe_7_(CN)_18_^3−^, has been tested on its biodegradation potential towards the selected cyano-based IL anions. This bacterial culture called KS-7D was investigated for the biodegradation of cyano-based anions in order to reduce their persistence and the risk of their accumulation in the environment (Fig. 1).

KS-7D is composed of the two bacterial species, *Cupriavidus basilensis* and *Cupriavidus eutrophus*, formerly *Ralstonia* spp. and *Wausteria* spp. (Vandamme and Coenye 2004). Both strains are capable of biodegrading cyanide and cyano–metal complexes. Free cyanide is hydrolysed by the bacteria to ammonia and formic acid (Bryniok and Trösch 2008), which is one of the metabolic pathways that can be used for the degradation of cyanide and cyano-based compounds (Gupta et al. 2010). This step is catalysed by a cyanide hydrolase without releasing the hazardous intermediate formamide and occurs independently of the presence of molecular oxygen and other cofactors. Ammonia and formic acid also serve as sources of nitrogen and carbon for the bacteria. The optimal growth conditions for the mesophilic KS-7D are temperatures between 30 and 40 °C and neutral to slightly basic pH of 7.5 to 8.5. These bacteria can also biodegrade ferrocyanide Fe(CN)_6_^4−^, ferric cyanide Fe(CN)_6_^3−^, and Prussian blue (Fe(III)_4_[Fe(II)(CN)_6_]_3_), which is one of the most stable cyano complexes. Cyanide concentrations of up to 1.4 g L^−1^ are still tolerated by KS-7D and no toxic effects are observed below a cyanide concentration of 6.5 mg L^−1^. This makes biodegradation a potential replacement for physico-chemical processes in the clean-up of cyanide-contaminated process effluents. The successful lab-scale application and the use of KS-7D as a starter culture for full-scale plants for cleaning waste air from flame lamination in the textile industry as well as the use of other similar bacteria in the biotreatment of cyanide wastes have already been reported (Dash et al. 2009; Sallat and Mählmann 2011). The KS-7D bacteria are microaerophilic betaproteobacteria strains isolated from a former coking plant site. They are niche bacteria that can be found in “soil, root nodules, aquatic environments, and human clinical sources” (Cserháti et al. 2012) with properties that are advantageous for bioremediation purposes. The complete genome of the *C. basilensis* OR16, isolated from a Hungarian pristine soil sample, has recently been sequenced due to the large xenobiotic-degrading potential of the *Cupriavidus* genus (Cserháti et al. 2012). Apart from the biodegradation of PB, the selected *Cupriavidus* spp. have successfully been investigated for other bioremediation purposes, such as the biodegradation of kraft lignin (Shi et al. 2013), the removal of toxic fermentation inhibitors, e.g. 5-hydroxymethyl furfural (Wierckx et al. 2010), the degradation of bisphenol A (Fischer et al. 2010), the removal of chlorophenols (Zilouei et al. 2006), the degradation of *s*-triazine (Stamper et al. 2002) and finally for the biodegradation of the first xenobiotic investigated, 2,6-dichlorophenol, to be degradable by the selected bacteria (Steinle et al. 1998). The bacteria in these studies have mainly been isolated from environments near sites that are contaminated with the corresponding chemical.

The use of axenic cultures for the biodegradation of a wider range of ILs has been successful, e.g. using *Sphingomonas paucimobilis* (Abrusci et al. 2011) or *Corynebacteria* for the biodegradation of pyridinium-based ILs (Zhang et al. 2010). In the present study, however, KS-7D did not biodegrade cyano-based IL anions, and changes made to the growth conditions of the bacteria did not improve the result either. Therefore, the question arose whether the bacteria may not biodegrade the anions because they use a combination of siderophores and cyanide hydralase for the biodegradation of PB; but no other nitrile hydrolysing enzyme, such as nitrilase or nitrile hydratase, had been reported. Since we know from abiotic hydrolysis studies on the cyano-based IL anions that at least N(CN)_2_^−^ and C(CN)_3_^−^ were hydrolytically instable under harsh pH conditions, we investigated whether the nitrile hydrolysing enzymes and commercially available nitrilase and nitrile hydratase may catalyse the hydrolysis as it has been shown several times for nitrile-containing chemicals (Martínková and Kren 2010; O’Reilly and Turner 2003) and could therefore be a hint for future bacteria selection for the biodegradation of cyano-based anions.

## Materials and methods

### Precultivation of bacteria in liquid medium

Concentrated culture of KS-7D (50 mL) was provided by the Fraunhofer Institute for Interfacial Engineering and Biotechnology, Stuttgart (Fraunhofer IGB Stuttgart, Germany). 40 mL of it was split into 1 mL aliquots. The remaining 10 mL were used for the first inoculation of the media. The medium composition recipe for the cultivation of KS-7D was followed as provided by Fraunhofer IGB Stuttgart. The analytical grade salts for the media were obtained from Sigma-Aldrich (Germany). The medium components were buffered using PBS at pH 7.5 (180 mg L^−1^ KH_2_PO_4_, 78 mg L^−1^ Na_2_HPO_4_*2H_2_O), 1 g L^−1^ MgSO_4_*7H_2_O, 2 mg L^−1^ Fe(III)-citrate and trace element solution SL4 (0.3 mg L^−1^ H_3_BO_3_, 0.1 mg L^−1^ ZnSO_4_*7H_2_O, 0.2 mg L^−1^ CoCl_2_*6H_2_O, 0.03 mg L^−1^ MnCl_2_*4H_2_O, 0.01 mg L^−1^ CuCl_2_*2H_2_O, 0.02 mg L^−1^ NiCl_2_*6H_2_O, 0.03 mg L^−1^ Na_2_MoO4*2 H_2_O). Fructose (C_6_H_12_O_6_, 0.72 g L^−1^) and potassium cyanide (KCN, 6.5 mg L^−1^) were specially added for the growth of the cyanide-degrading bacteria.

The bacteria were used for the experiments after 2 days of incubation in order to ensure optimal cell numbers of 10^4^–10^5^ in 100 mL. For the cultivation of KS-7D, 50 mL of grown culture were added to 950 mL of medium. The amount of left KCN was checked via Hach Lange cuvette test LCK315 (0.01–0.6 mg L^−1^ CN^−^) and was below the quantification limit. The bacteria were kept in an incubator (A120S–LAUDA Dr. R. Wobser GmbH & Co. KG, Germany) at a temperature of 30 °C, where they were gently and continually shaken horizontally at medium speed.

The bacteria from an industrial waste water treatment plant (Merck KGaA factory, Darmstadt, Germany) were not cultivated but received from the plant directly and used within a limited time period of one week. It was taken there, transported in a 10-L container overnight and aerated with oxygen in our lab before use. The industrial activated sludge is not expected to contain the KS-7D bacteria strains.

### Cell counter measurement of the bacterial growth

The bacterial growth in the medium composition recipes was examined using cell counter (Z SERIES COULTER COUNTER®, Beckmann Coulter Electronics GmbH, Germany). The particle size range was set to 1.7–7.8 μm. The bacterial growth of each cyano-based ILs (at a concentration of 100 mmol^–1^) was investigated in the presence of fructose. The measurements were conducted four times for each sample after 0, 4, 6 and 24 h of cultivation. One millilitre of bacteria suspension was added to 10 mL of isotonic solution (COULTER® ISOTON® II diluent). The instrument was flashed with isotonic solution three times before each measurement.

### The experiments

The question whether IL anions are biodegradable by KS-7D or, if not, can be hydrolysed by isolated enzymes has been addressed with different experimental conditions which are summarised in Tables 1 and 2. A more detailed description can be found in the following paragraph:

**Table 1:** Overview on the biodegradation tests conducted in this study

Test no.	Type	Analyte	Comment
A	Biodegradation tests on agar plates	Prussian blue	Investigation on the effective operation of the used bacteria strains; duration: 48 h; mode of detection: loss of blue colour
B	Biodegradation tests in liquid medium	IL anions	Tests using conditions which are increasingly preferred by the used bacteria; duration: 28 days; mode of detection: specific analysis of the anion via ion chromatography
C	Enzymatic degradation test	IL anions	Investigation on the stability of the anions in the presence of nitrile degrading enzymes; duration: 24 h; mode of detection: specific analysis of the anion via ion chromatography and mass spectrometry

**Table 2:** Overview on the changing test conditions used for the biodegradation test B

Test no.	Medium	Temperature (°C)	Bacteria medium ratio (%)	C/N ratio	Type of bacteria
B1	Stringent test medium “OECD guideline 301 medium (OECD 1992)”	20	1	5	KS-7D
					Activated sludge (industrial waste water treatment plant)
B2	Optimised test medium for KS-7D “cultivating medium from Fraunhofer IGB Stuttgart”	30	10	10	KS-7D
B3	Nutrient-rich medium “same medium composition as for the agar plates, but without agar, so that the medium remained liquid. This medium consisted of peptone, yeast extract and sodium chloride”	20	1	5	KS-7D

A. Biodegradation tests on agar plates
  At first the bacteria were incubated on agar plates in order to examine the biodegradation of the positive control PB. Twelve agar plates were set up and PB was added to half of the plates before the agar solidified. Two pairs of PB/non-PB plates were inoculated with (1) pure culture of KS-7D and (2) bacteria from an industrial waste water treatment plant. The remaining pair was not incubated and served as a blind control. The plates were incubated in the dark at 37 °C for 48 h.
B. Biodegradation tests in liquid medium
  To examine the biodegradation of the IL anions, the experiment was set up at different growth conditions. The biodegradation experiments were run in 100 mL autoclaved glass vessels. As a blank for the ion chromatographic detection of the IL anions, one vessel was set up that contained medium and bacteria only, without the analyte. For each IL two vessels were used. IL with the amount of 100 μmol L^−1^ was added to the medium and bacteria. The total C/N ratio, adjusted by the addition of fructose, is related to the concentration of the carbon and nitrogen in the medium and the different IL anions. Thereby, the C/N ratio is the carbon to nitrogen ratio of the total of substrates: IL anion and fructose, if necessary.
  Different media were used for the experiments: (1) OECD guideline 301 medium (OECD 1992), (2) cultivating medium from Fraunhofer IGB Stuttgart and (3) nutrient-rich medium, with different experimental conditions. The first experiment on the biodegradability of IL anions was run at a temperature of 20 °C with 1 % bacteria suspension and a C/N ratio of 5 of the total of substrates (IL anion and fructose). KS-7D and bacteria from an industrial waste water treatment plant were used. In the second run of the first experiment, the medium was enriched with more bacteria (10 %), with a higher C/N ratio of 10 and a higher temperature of 30 °C. In the second experiment, the enriched conditions in terms of bacteria content (10 %), C/N ratio of 10 and temperature of 30 °C were maintained and, this time, applied within the original cultivating medium from Fraunhofer IGB Stuttgart. The KCN in this recipe was substituted with a CN-containing IL. For a comparable medium composition of the liquid medium experiment and the agar plates, the third experiment was run with the same medium composition as for the agar plates, but without agar, so that the medium remained liquid. This medium consisted of peptone, yeast extract and sodium chloride. The temperature of 20 °C and C/N ratio of 5 were applied. One experiment run took 28 days. In the first 7 days, samples were taken daily and thereafter once a week on days 14, 21 and 28. The samples for the ion chromatographic measurement were centrifuged (RCF 1700, 15 min) and passed through RC-filters (ROTH®). The ion chromatograph (IC) used was an “IC Metrohm 881 Compact pro” (Metrohm, Switzerland) with a “Metrosep A Supp 5” anion exchanger column. The device was run at a flow rate of 0.7 mL min^−1^ and an injection volume of 20 mL. The standard IC eluent for anions (3.2 mmol L^−1^ Na_2_CO_3_, 1 mmol L^−1^ NaHCO_3_) was modified with acetonitrile to enhance the detection of the larger and more lipophilic anions C(CN)_3_^−^ and B(CN)_4_^−^. Limit of detection and limit of quantification were below 0.1 and 0.3 μmol L^−1^, respectively.
C. In vitro enzymatic hydrolysis
  To examine the in vitro enzymatic hydrolysis of the selected IL anions, two enzymes were used that are able to catalyse the reaction: (1) nitrilase and (2) nitrile hydratase. Both enzymes are recombinants from *Escherichia coli* and purchased from Sigma-Aldrich Chemie GmbH, Steinheim, Germany (CAS numbers (1) 9024-90-2; (2) 82391-37-5). The experiment was conducted according to common nitrilase reactions in organic synthesis (Banerjee et al. 2009; Rey et al. 2004; Robinson and Hook 1964). The reaction medium was therefore a 100-mM KH_2_PO_4_/K_2_HPO_4_ buffer at pH 7.5 (pH adjustment with KOH). All salts were obtained from Merck KGaA, Darmstadt, Germany. The concentration of the IL anion and the enzyme was 1 mmol L^−1^ and 4 g L^−1^, respectively. Three control samples were prepared in duplicates in 1.5 mL Eppendorf cups: (B1) IL anion in buffer, (B2) nitrilase in buffer, and (B3) nitrile hydratase in buffer. IL anion and nitrilase in buffer and IL anion and nitrile hydratase were also prepared. Additionally, three control samples (without enzyme) were arranged. Half of these samples were further prepared for ion chromatographic analysis. The other half was placed in an Eppendorf AG Thermomixer compact at 35 °C and the lowest speed of 300 rpm overnight (ca. 22 h).

### Sample preparation for ion chromatography

One millilitre of the sample was transferred into a 15-mL polypropylene centrifuge tubes (Sarstedt AG & Co., Germany). The proteins could then be precipitated by addition of acetone 1:4 (HiPerSolv CHROMANORM for HPLC, VWR International). The centrifuge tubes were then placed into crushed ice for 20 min. The samples were then centrifuged at 3,000 rpm for 10 min which makes around 1,200 g (Labofuge 400R, Heraeus Instruments GmbH, Germany). The supernatant was transferred to round bottom flask and the solution was rotary evaporated in a water bath at 40 °C and vacuum (VV2011, Heidolph Instruments GmbH & Co.KG, Germany). The final residue was diluted in 10 mL of deionised water, giving the final concentration of 100 μmol L^−1^ for the ion chromatographic analysis. Final sample (500 μL) was diluted 1:1 in methanol (HiPerSolv CHROMANORM for HPLC, VWR International) for mass spectrometric analysis. The mass spectrometer was an esquire ESI-MS with ion trap detector (Bruker Daltonik GmbH, Germany). The samples were directly injected using a syringe pump at a flow rate of 3 μL/min. The nebuliser was run at 5 psi, with a dry gas flow rate of 5 L min^−1^ and a drying temperature of 300 °C. The capillary voltage was set to +4,000 V. The anions were detected using the negative mode.

## Results and discussion

### Biodegradation of the positive control on agar plates

**Fig. 2:**
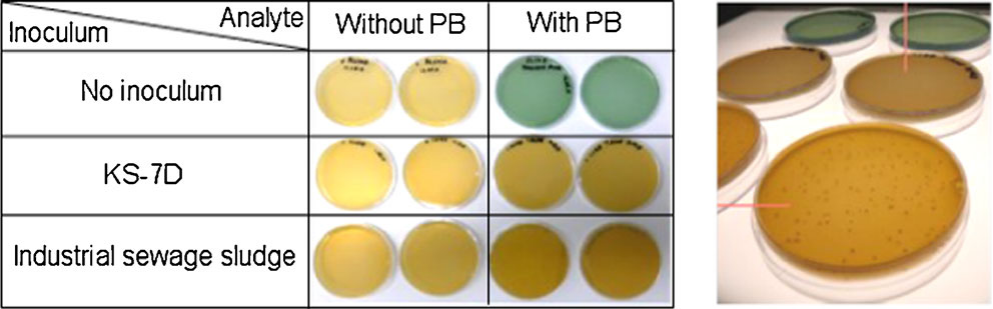
Left: results of the biodegradation of Prussian blue (PB) in agar plates by different inocula. Right: close-up of the bacteria colonies

The biodegradation of PB was investigated to find out whether the KS-7D bacteria would degrade the analyte as expected and if there was a difference in comparison to industrial sewage sludge. Agar plates were used, since the PB was agglutinating in the liquid medium, forming blue particles, whereas in agar it remained diluted. It could be seen that with both KS-7D and industrial sewage sludge, the blue colour of the PB agar reverted to the original colour of the agar. The blind control without inoculum remained blue, showing that without inoculation the analyte remained stable during the incubation time of 2 days (Fig. 2).

The results do not match the hypothesis. That KS-7D would degrade PB was expected, but it was not expected that the industrial sewage sludge would do the same, since not many such cases have been reported for metal cyanide complexes (Wehrer et al. 2011). Only a few microorganisms are known to grow on iron cyanide complexes by converting the cyanide into carbon dioxide and ammonia as nitrogen source, among them some *Acinetobacter* spp. (Finnegan et al. 2000) and *Pseudomonas* spp., such as *Pseudomonas fluorescens* (Dursun et al. 1999) and *Pseudomonas pseudoalcaligenes* (Luque-almagro et al. 2005). Additionally, fungi like the filamentous *Fusarium solani* and *Fusarium oxysporum* have shown to grow on iron and nickel cyanide complexes at normal to acidic pH values (Barclay et al. 1998a). The fungi also hydrolyse the cyanide to ammonia, which serves as nitrogen source, and formate (Barclay et al. 1998b). PB degradation could further be observed in the rhizosphere of cyanogenic plants again with the help of microbial transformation (Kang et al. 2007). Next to the cyanide degradation, the decolouration of PB could also be caused by bacterial reduction of Fe^3+^ ions of the outer complex sphere and not necessarily due to the degradation of the Fe(CN)_6_^−^ moiety. Such a mechanism has been described for the iron-reducing fresh water and marine bacteria *Geobacter metallireducens* and *Shewanella alga* strain BrY, respectively. These bacteria use PB as sole electron acceptor in iron respiration (Jahn et al. 2006). Whether any of these microorganisms have been involved in the decolourisation of the agar plates inoculated with industrial sewage sludge was not examined. The disappearance of the blue colour in our study, however, proves the biological activity of the used bacteria.

### Bacterial growth in the presence of KCN and cyano-based ILs

**Fig. 3:**
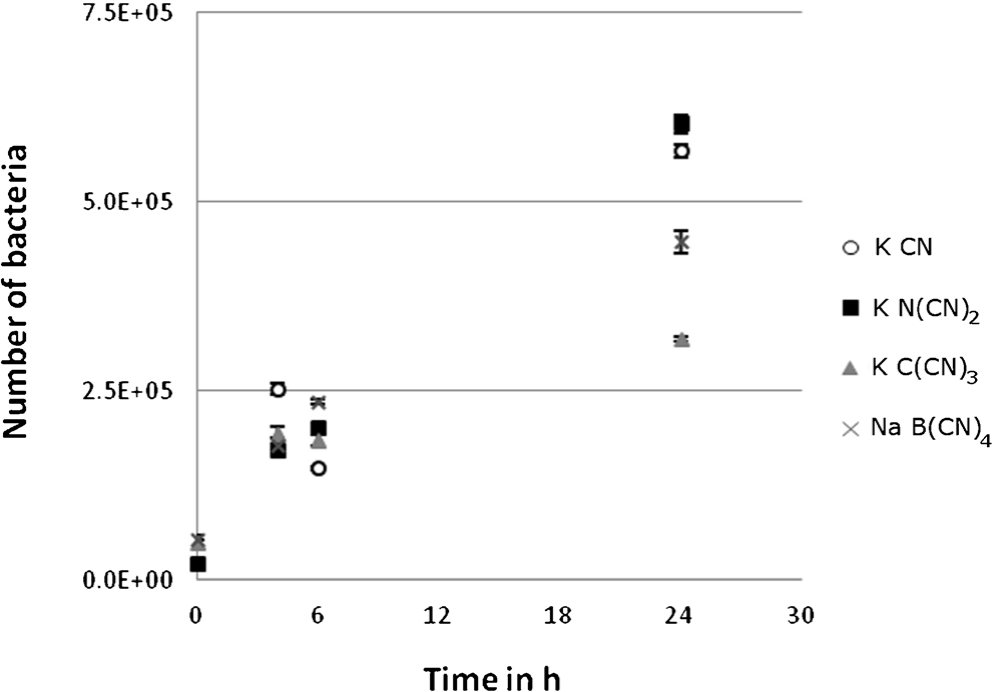
Number of KS-7D bacteria over time cultivated in medium based on the Fraunhofer IGB recipes containing KCN, K N(CN)_2_, K C(CN)_3_ and Na B(CN)_4_, respectively

Recent studies on the toxicity of N(CN)_2_^−^ and B(CN)_4_^−^ towards activated sludge revealed a higher inhibition potential for B(CN)_4_^−^ than for N(CN)_2_^−^ (Markiewicz et al. 2013; Neumann et al. 2012). We tested the bacterial growth of KS-7D in presence of selected cyano-based ILs (100 μmol L^−1^). In this study the growth of the bacteria via cell counter measurements was followed during 24 h (Fig. 3). N(CN)_2_^−^ showed no inhibition, whereas for C(CN)_3_^−^ and B(CN)_4_^−^, a slight inhibition of bacterial growth was observed compared to KCN. The KS-7D bacteria themselves were able to grow significantly under the experimental conditions in the presence of all selected IL anions. Based on these findings false-negative results, with the lack of biodegradation being the effect of the IL's toxicity towards bacteria, are not assumed.

### Biodegradation of the cyano-based ionic liquid anions

**Fig. 4:**
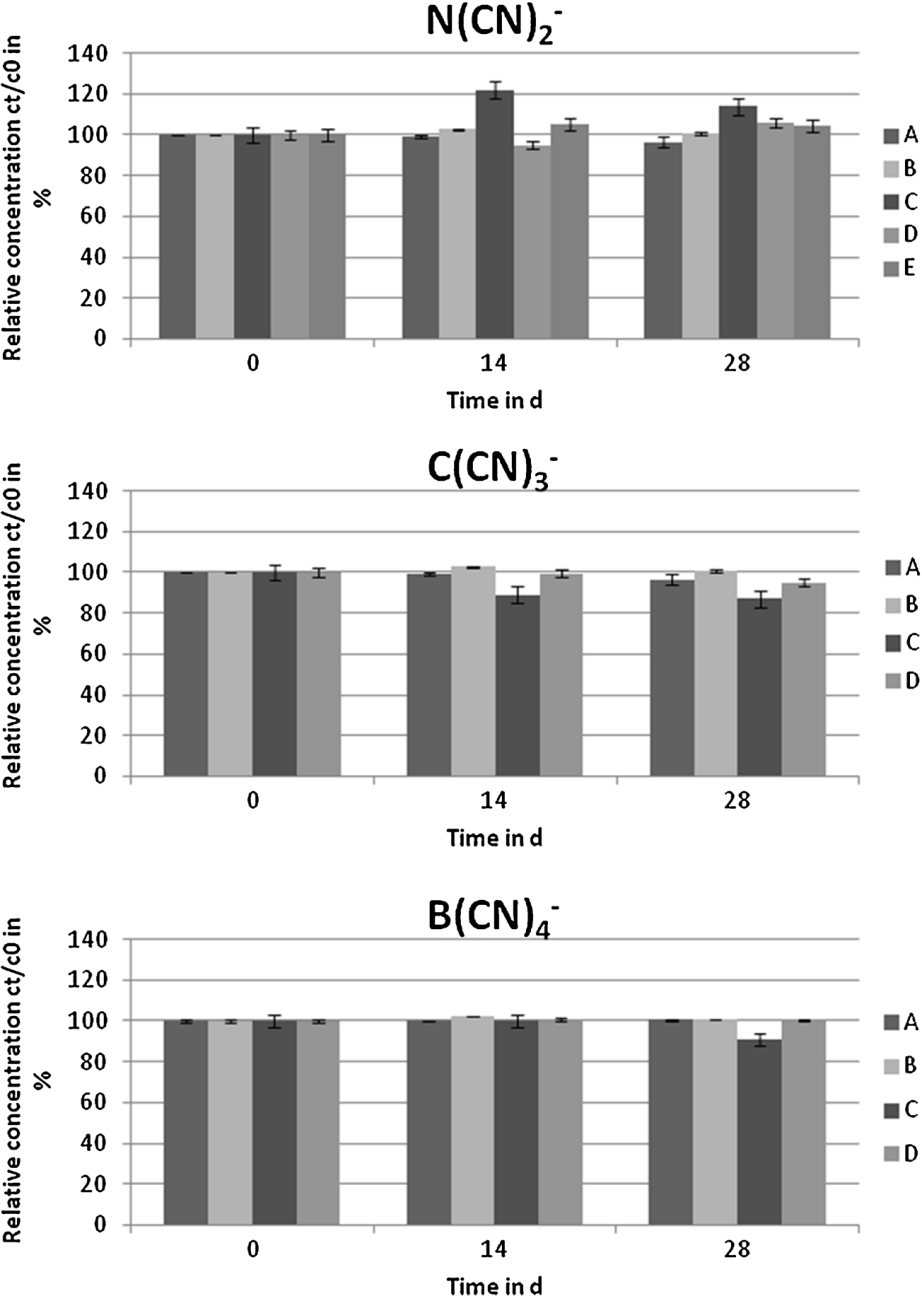
Relative concentration of N(CN)_2_^−^, C(CN)_3_^−^ and B(CN)_4_^−^ on different days of the biodegradation test and test conditions: (*A*) KS-7D bacteria and (*B*) industrial sewage sludge bacteria (*T* = 20 °C, OECD test guideline 301 medium, 1 % bacteria suspension, C/N ratio 5), (*C*) KS-7D bacteria with an enriched medium (*T* = 30 °C, OECD test guideline 301 medium, 10 % bacteria suspension, C/N ratio 10), (*D*) KS-7D bacteria with an enriched medium (*T* = 30 °C, Fraunhofer IGB Stuttgart medium, 10 % bacteria suspension, C/N ratio 10) and (*E*) KS-7D bacteria with agar medium composition (*T* = 20 °C, liquid agar medium, 1 % bacteria suspension, C/N ratio 5)

Different growth conditions for the KS-7D bacteria were chosen to enhance their biological activity, and the biodegradability of the cyano-based anions was determined via specific analysis. None of the conditions have a significant decrease in relative analyte concentration observed: either at a temperature raised from 20 to 30 °C, together with an elevated bacteria and C/N content (Fig. 4 A, B vs. C, D), or using the same media composition as with the earlier agar plate test (Fig. 4 E). Neither the KS-7D nor industrial sewage sludge bacteria (Fig. 4 A and B) were able to use the anions as carbon or nitrogen sources.

Without a decrease in relative concentration, the anions are stated to be not primarily biodegradable, where primary biodegradation is the first step in the biodegradation of the whole compound. The difference in relative concentration to up to ±10–20 % is related mainly to measurement uncertainties that are high in biological degradation tests, especially when matrices with a high organic load are used. The non-biodegradability of cyano-related compounds was also observed in another study in which iron cyanide complexes were investigated for their biodegradability by the addition of cyano-degrading bacteria (Oelsner et al. 2001). The explanation for this is that the bacteria de-adapt as soon as the carbon and nitrogen levels in other components of the medium suffice as nutrients, so that the bacteria no longer need the cyano complexes for their growth. Since the bacteria mixture KS-7D is reported to require an additional carbon source for exponential growth (Bryniok and Trösch 2008), this explanation appears to be unlikely. It must further be considered that the KS-7D bacteria are able to use ferrocyanide, ferric cyanide and PB not only as carbon and nitrogen source, but as a source of iron, too, and release siderophores (small iron-binding molecules) into the medium to cleave these complex iron cyanides (Schygulla-Banek 1993). It seems that the cyanide hydrolase of KS-7D is now not capable to cleave the C ≡ N bond as long as the cyanide is covalently ligated. No molecules, such as the siderophores, seem to exist in KS-7D that can cleave the bond between the central atoms B, C and N, respectively, and the carbon of the cyano groups of the ILs. At the moment, no organism has been found that biodegrades cyano-based IL anions.

### In vitro enzymatic hydrolysis

We investigated if cyano-based anions are generally susceptible towards in vitro enzymatic hydrolysis using a commercially available nitrilase (NLase) and nitrile hydratase (NHase). Such enzymes are used as catalysts in organic synthesis for the hydrolysis of nitrile groups in pharmaceutical industry and for bioremediation purposes amongst others (Banerjee et al. 2002; Kobayashi and Shimizu 1998; Mascharak 2002; Singh et al. 2006).

**Fig. 5:**
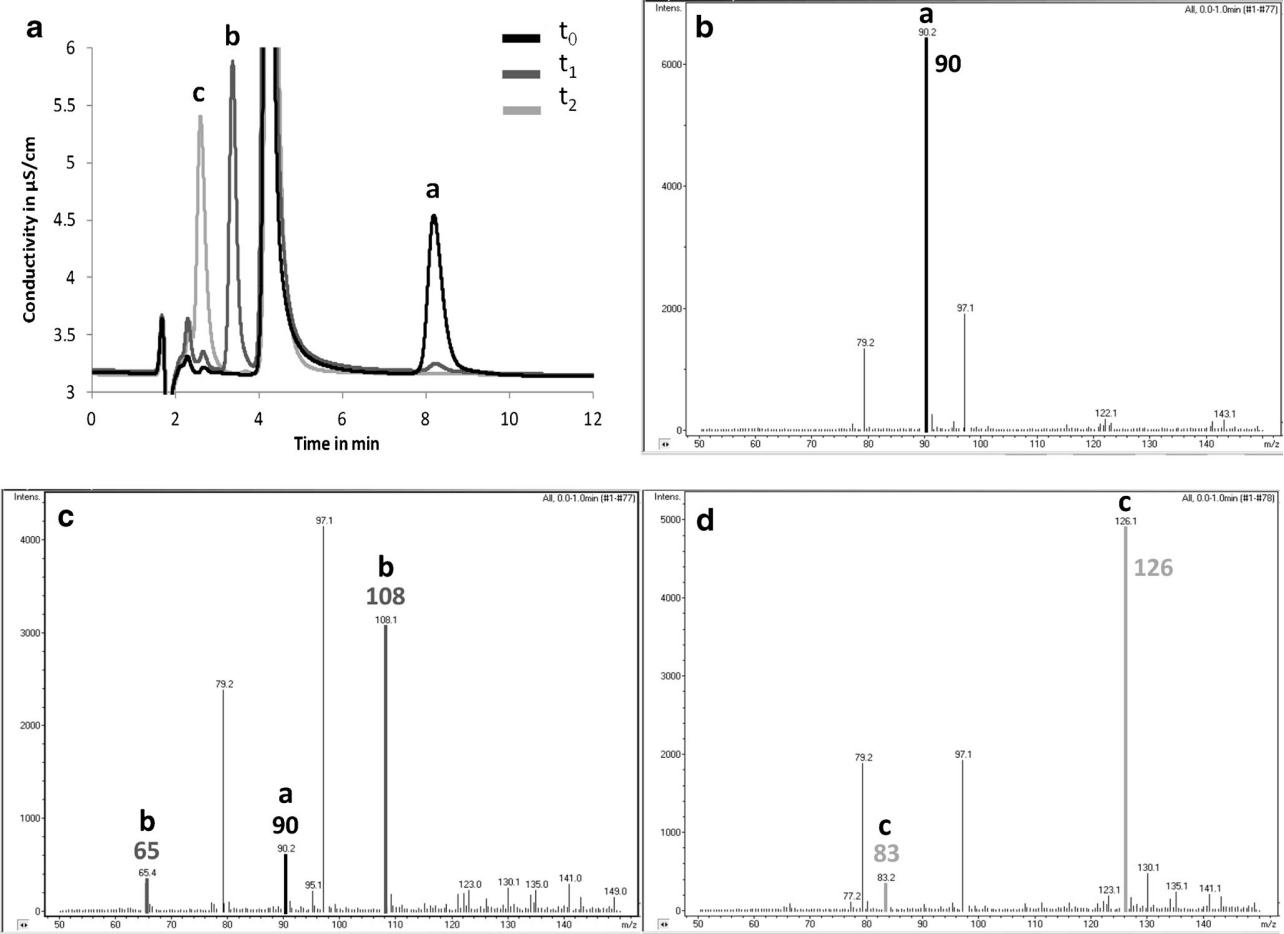
Results of the in vitro enzymatic hydrolysis of C(CN)_3_^−^ with NHase. **a** Ion chromatogram overlay at three different points of time in the experiment: *t*_0_ without enzyme treatment (*t* = 0 min); *t*_1_ immediately after enzyme addition (*t* = 1 min); *t*_2_ with enzyme treatment (*t* = 22 h). **b**–**d** Corresponding mass spectra from *t*_0_ to *t*_2_. The compounds detected were (*a*) C(CN)_3_^−^_,_ (*b*) C(CN)_2_(CONH_2_)^−^ and the MS-fragment C(CN)_2_H^−^, (*c*) C(CN)(CONH_2_)_2_^−^ and the MS-fragment C(CN)(CONH_2_)H^−^

In experiments with NLase, the concentration of all anions remained stable within 22 h and the experimental uncertainty levels of around 10 %, and no transformation products were found. In contrast, all of the cyano-based anions were hydrolysed to different extent by NHase and corresponding amides that could be detected (Table 3). The analytical results are exemplified using the example of C(CN)_3_^−^ (Fig. 5).

**Table 3:** Table of the detected anions (a–h), their formula, the net retention times (*t*_r_) and concentrations in ion chromatographic analysis and the corresponding mass-to-charge ratios visible in the mass spectra of the mass spectrometer of the detected IL anion and IL anion transformation products

Anion	Labels in Figs. 5 and 6	Formula	*t*_r_ (min)	Relative concentration (c_0_/c_t_) (%)						Mass-to-charge ratio *m*/*z* of the IL anion related peaks in mass spectra	
				NLase			NHase			M^−^	[M-43]^-^
				*t*_0_	*t*_1_	*t*_2_	*t*_0_	*t*_1_	*t*_2_	Molecular ion peak	Fragmentation: −HNCO via hydrogen rearrangement
Dicyanamide	a	N(CN)_2_^−^	7.2	100	101	103	100	76	n.d.	66	–
	b	N(CN)(CONH_2_)^-^	0.9	n.d.	n.d.	n.d.	n.d.	15	66	84	n.d.^a^
Tricyanomethanide	c	C(CN)_3_^−^	6.3	100	104	101	100	3	n.d.	90	–
	d	C(CN)_2_(CONH_2_)^−^	1.6	n.d.	n.d.	n.d.	n.d.	89	n.d.	108	65
	e	C(CN)(CONH_2_)_2_^−^	0.8	n.d.	n.d.	n.d.	n.d.	3	67	126	83
Tetracyanoborate	f	B(CN)_4_^−^	10.7	100	101	102	100	99	6	115	–
	g or h	B(CN)_3_(CONH_2_)^−^	1.7	n.d.	n.d.	n.d.	n.d.	n.d.	5	133	90

*t*_0_ without enzyme treatment (*t* = 0 min), *t*_1_ immediately after enzyme addition (*t* = 1 min), *t*_2_ with enzyme treatment (*t* = 22 h), *n.d*. not detected

^a^Scan range *m*/*z* 50–200

**Fig. 6:**
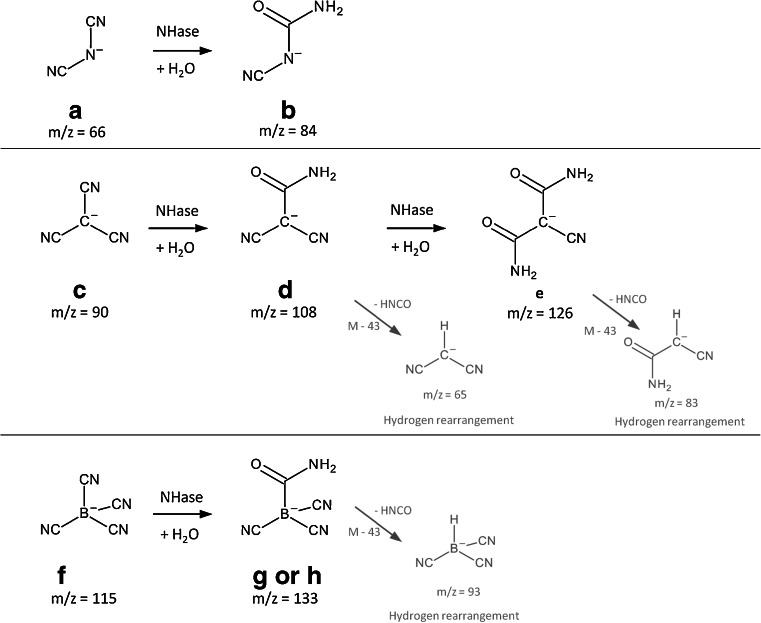
Proposed in vitro hydrolytical pathway by NHase at pH 7 of the investigated IL anions together with the mass-to-charge ratios (*black*) and fragmentation pattern detected via mass spectrometry (*grey*)

The sample that was taken immediately after the NHase addition (*t*_1_) already shows a degradation of the parent compound in comparison to the sample without any enzyme addition (*t*_0_). A transformation product that could be identified via MS as the C(CN)_2_(CONH_2_)^−^ anion appears just before the phosphate buffer peak at 4.5 min in the IC chromatogram. After 22 h this transformation product disappeared and the next hydrolytical product could be detected, C(CN)(CONH_2_)_2_^−^. A similar behaviour has been observed for N(CN)_2_^−^ and B(CN)_4_^−^, where they have been hydrolysed into N(CN)(CONH_2_)^−^ and B(CN)(CONH_2_)^−^, respectively. The proposed hydrolytical transformation pathway for the IL anions is shown in Fig. 6.

The observations revealed that a hydrolysis under pH neutral conditions of the IL anions by NHase leads to the corresponding amides. Even B(CN)_4_^−^ which is stable under harsh conditions could be transformed. The non-hydrolysis by NLase within the experimental run time is assumable due to the completely different mechanisms in NLase and NHase. NLase attacks the C-atom of the nitrile group to form a covalently bonded thiomidate intermediate. This thiomidate is further oxidised and the corresponding carboxylated product is formed. In NHase no such covalent intermediate needs to be formed. There, either the cyano group of the substrate is directly attacked by a metal-bound hydroxide ion acting as a nucleophile or the metal-bound hydroxide ion acts indirectly as a base to first activate a water molecule, which then attacks on the cyano group (Banerjee et al. 2002). The nitrile is finally hydrolysed into the amide. Therefore, it is assumed that the NHase can more easily access the cyano-based anions. For the degradation of cyano-based IL anions, it appears promising to use other NHase-containing microorganisms such as *Rhodococcus erythropolis* which is capable to degrade e.g. benzonitrile herbicides (Veselá et al. 2012).

## Conclusions

The overall aim of the experiments was to find out whether cyano-based anions can be degraded with the aid of KS-7D bacteria for potential application in waste water treatment or further by nitrile-hydrolysing enzymes. At the moment no biodegradation of the cyano-based anions has been observed by the used microorganisms. The capability of nitrile hydratase to hydrolyse the cyano-based anions in vitro now leads to further considerations. Since pure enzymes are relatively instable, they are often more applicable in organic synthesis than wastewater treatment. An alternative may be cross-linked enzymes aggregates (CLEAs) that are made of immobilised and stabilised enzymes and are recycable for multiple uses (Sheldon 2011). The investigated NHase has been especially successfully prepared as CLEAs for “green nitrile hydration in industry” (van Pelt et al. 2008). The use of CLEAs of laccase is also currently under investigation for use as potential treatment procedure for waste water contaminated with endocrine disruptors (Wintgens 2013). Another alternative to the pure enzymes used could be the direct use of bacteria that contain the necessary enzymes for nitrile hydrolysis, such as *R. erythropolis* (Vejvoda et al. 2007). In general, bacteria could be advantageous to pure enzymes. They contain a series of different enzymes that can make amides not only from nitriles but also carboxylic acid from the produced amides by amidases, and even a complete mineralisation may be further realisable. Whether or not any of the mentioned possibilities are applicable for the treatment of wastewater that is contaminated with cyano-based anions will then need to be tested. In terms of a structural design of IL anions, the synthesis of new anions that have an intrinsically higher potential for being biodegradable should also be considered. For example in the case of tetracyanoborate, the borate could already be prepared as a carboxylated anion, e.g. B(CO_2_H)_4_^−^ (Bernhardt et al. 2006), and a hydrogenated one, e.g. BH_2_(CN_2_) (Györi et al. 1983), as well as a dianion B(CN)_3_^2−^ (Bernhardt et al. 2011) that could change the accessibility of the molecule for enzymatic attack.
